# New-Onset Seizures as an Acute Presentation With Atypical EEG Findings in a Previously Healthy Child With Asymptomatic COVID-19 Infection

**DOI:** 10.7759/cureus.22899

**Published:** 2022-03-06

**Authors:** Asra Akbar, Sharjeel Ahmad

**Affiliations:** 1 Pediatric Neurology, University of Illinois College of Medicine at Peoria, Peoria, USA; 2 Infectious Diseases, University of Illinois College of Medicine at Peoria, Peoria, USA

**Keywords:** covid-19, sars-cov-2, electroencephalography (eeg), eeg, status epilepticus (se)

## Abstract

Coronavirus disease 2019 (COVID-19) infection is caused by severe acute respiratory syndrome coronavirus 2 (SARS-CoV-2). This infection usually presents with upper respiratory symptoms; however, it can also present with a wide variety of other multisystem and neurological symptoms, including seizures. There are several proposed mechanisms by which COVID-19 can cause systemic signs of infections, including neurological complications and seizures.

This case report describes a pediatric patient without a previously documented history of epilepsy who was admitted for new-onset focal seizures with impaired consciousness. No other cause and triggers of seizures were found, and the child was tested positive for COVID-19 infection. The patient had six electroclinical seizures during EEG. Video EEG findings showed atypical features of onset of intermittent rhythmic delta activity (IRDA) slowing over the left hemisphere with evolution/generalization of rhythmic delta/theta activity and without clear typical generalized epileptiform discharges. These EEG findings correlated with a clinical change of behavior arrest, staring, and yawning. Similar spells were reported multiple times a day prior to the admission, and past EEG was normal. A review of current literature on COVID-19 and neurological manifestations in children, including new seizures and prior diagnosis of epilepsy, is also provided in this case report.

The clinical experience in children with newly diagnosed or chronic epilepsy suggests that exacerbation of seizures, especially from systemic effects such as those caused by severe COVID-19 infection, will be a major concern.

## Introduction

Coronavirus disease 2019 (COVID-19) is caused by severe acute respiratory syndrome coronavirus 2 (SARS-CoV-2). This infection usually presents with upper respiratory symptoms; however, it can also present with a wide variety of other multisystem and neurological symptoms, including seizures.

There are several proposed mechanisms by which COVID-19 can cause systemic signs of infections, including neurological complications and seizures. These mechanisms include direct infection, autoimmune response, postinfectious process, and vascular processes, including thrombosis and infarction [1]. Dysregulation of cytokine signaling is also a proposed mechanism. Proinflammatory cytokines such as interleukin (IL)-6, interferon-γ, and IL-8 are associated with febrile seizures [2]. Cerebrospinal fluid IL-6 has been reported to have an association with complex febrile seizures [3].

The clinical experience in children with newly diagnosed or chronic epilepsy suggests that exacerbation of seizures, especially from systemic effects such as those caused by severe COVID-19 infection, will be a major concern.

## Case presentation

A seven-year-old previously healthy child was admitted for spells of loss of awareness that lasted about 10-15 seconds, which had increased in duration and frequency since onset over a year ago. During these episodes of behavior arrest, she would also yawn and these episodes were associated with the ictal EEG findings of intermittent rhythmic delta activity (IRDA) slowing over the left hemisphere with evolution/generalization of rhythmic delta/theta activity and without clear typical generalized epileptiform discharges.

The parents denied fever, vomiting, headaches, neck stiffness, lightheadedness, blurry vision, difficulty with speech, weakness/numbness/tingling in arms or legs, changes in bowel or bladder function, or recent trouble with gait or falls. There was no prior history of traumatic brain injury, febrile seizures, family history of epilepsy, and/or a prior documented history of epilepsy.

Prior to the admission, she had other episodes of "legs being weak" and falls over a few months. The mother had reported multiple spells a day of loss of awareness, and an EEG was obtained for characterization of these spells, which were seen daily and several times a day, which read as normal. The patient was evaluated by a child neurologist at that time, and these spells were thought to be syncopal events with a normal EEG. A diagnosis of epilepsy was not made prior to this admission months later. These staring spells increased in duration and were characterized by yawning and confusion as well as falls and loss of bladder control, and she was admitted for further evaluation. Birth history and developmental history were reported as normal.

On admission, a complete blood count (CBC), complete metabolic panel (CMP), and urine analysis (UA) were obtained, which were all normal. A respiratory pathogen array obtained for several viruses including adenovirus, influenza, parainfluenza, and respiratory syncytial virus (RSV) was negative. Cerebrospinal fluid (CSF) analysis for cell count, glucose, protein, and culture was normal and herpes simplex virus (HSV) polymerase chain reaction (PCR) did not show any evidence of CNS infection (Table 1).

**Table 1:** CSF analysis on admission.

Component	Value	Reference range	Units
CSF protein	14.9	12-60	mg/dl
CSF glucose	42	40-70	mg/dl
CSF cell count	0	0-5	/mm(3)
CSF lymphocytes			
CSF RBC	96	0	/mm(3)
CSF IgG		<8.1	mg/dl

SARS-CoV-2 nasopharyngeal swab by PCR testing was positive. SARS-CoV-2 IgG was positive (1.4 S/C). Chest X-ray was normal. C-reactive protein (CRP), procalcitonin, and D-dimer were normal.

**Figure 1:**
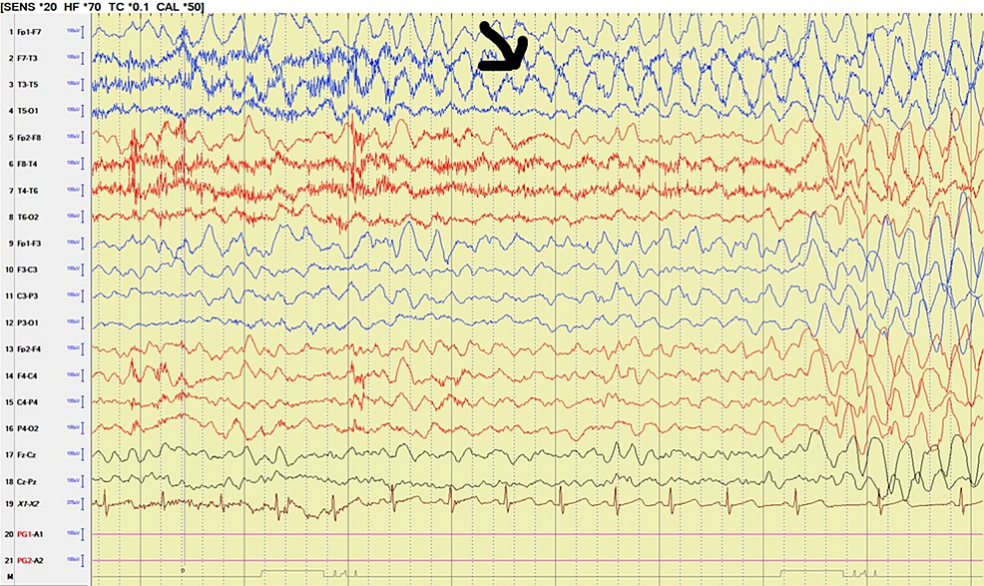
Video EEG findings showing the onset of left-sided rhythmic low theta slowing associated with a clinical change of behavior arrest (arrow).

**Figure 2:**
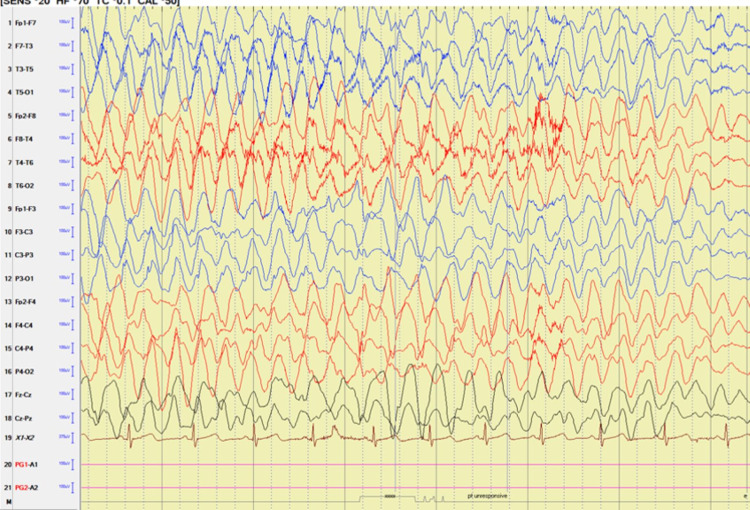
Video EEG findings with the evolution of generalized rhythmic delta slowing associated with a clinical change.

Video EEG captured six electroclinical seizures (Figures 1, 2). The review of the video showed a clinical change of yawning, confusion, and behavior arrest lasting 8-10 seconds. These spells were associated with loss of bladder control and loss of tone with resultant falls. Occasional finger rubbing and nose touching were noted at the end of the seizure.

On EEG, the clinical changes correlated with runs of low theta slowing with onset noted over the left temporal region that evolved to generalized high amplitude rhythmic delta slowing lasting 10-15 seconds in duration (Figures 1, 2).

**Figure 3:**
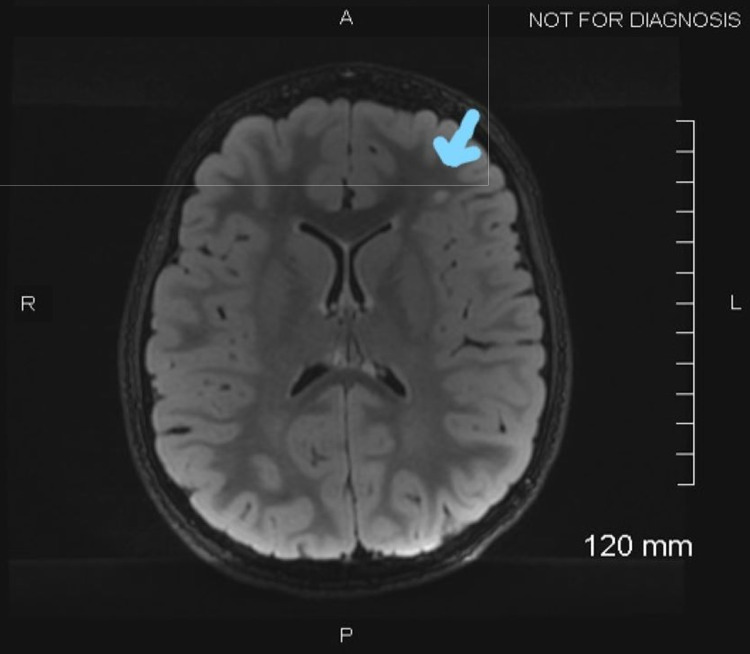
MRI of the brain showing T2 fluid-attenuated inversion recovery (FLAIR) multifocal subcortical white matter changes.

MRI of the brain with and without contrast showed several non-enhancing scattered T2 fluid-attenuated inversion recovery (FLAIR) subcortical white matter hyperintensities in the bilateral frontal lobes (Figure 3).

Chromosomal microarray, lactate, pyruvate, serum amino acid, and urine organic acid were normal. Epilepsy panel was sent to reference lab (GeneDx Laboratory, Gaithersburg, MD), which showed a variant of unknown clinical significance with dynamin 1 (DNM1) and SLC2A1 mutation. Both the parents were tested and the mother was negative. The father had the same gene of unknown clinical significance and was asymptomatic without a diagnosis of epilepsy.

She was discharged home on Keppra and since then seizures are well controlled. Repeat EEG was obtained four weeks later, which read as normal. On follow-up visits, the family reports intermittent seizures, which have responded to changes in medication dose with weight.

## Discussion

COVID-19 is caused by SARS-CoV-2 and was first detected in Wuhan, China, in December 2019. It can affect both the central and peripheral nervous system, and neurological manifestations have also been rarely reported in children. Anosmia, stroke cranial nerve deficits, meningitis, and seizures are neurological complications of COVID-19 infection. Seizures in COVID-19 patients have been first documented by Moriguchi et al. [4]. Neurological manifestations of COVID-19 with CNS symptoms are reported in 25% of patients but seizures were reported in only 0.5% [5].

Several viruses, including influenza, adenovirus, and RSV, have been known to cause seizures [6]. Evidence suggesting the ability of some coronaviruses to invade the brainstem suggests the possibility that SARS-CoV-2 has a similar ability to invade the CNS [7]. Coronaviruses can enter cells that express angiotensin-converting enzyme 2 (ACE2), which has been found to be expressed by neurons and glial cells [8].

Acute symptomatic epileptic seizures and status epilepticus (SE) are two of the neurological conditions most frequently reported in association with SARS-CoV-2 infection and carry a high rate of mortality (between 5% and 39%) [9]. However, the effect of COVID-19 infection on the evolution of newly diagnosed epilepsy is not well known. The patient’s epilepsy panel showed a variant of unknown clinical significance with DNM1 (c2372 C>GP A791G) mutation, which the father also had but was asymptomatic.

DNM1 is a large guanosine triphosphatase involved in clathrin-mediated endocytosis. DMN1 mutation is associated with intractable epilepsy, developmental delay, and hypotonia in pathogenic variants. The clinical significance is spurious in this case.

Our review of literature looking for acute onset seizures in the pediatric patients and COVID-19 infection (Table 2) using PubMed (National Institutes of Health, National Library of Medicine, USA) between November 2020 and July 2021 showed seven children who presented with acute onset seizures and SE and were tested positive for COVID-19 infection. The children were between the ages of day of life two to 13 years old. There were five male children and two female children. The most common presentation was generalized tonic-clonic seizures and SE. EEG findings were recorded in six out of the seven children. One patient's EEG showed electrographic seizures, one patient's EEG showed excessive sharp waves, two of the patient's EEG showed generalized slowing, one patient's EEG showed frontal intermittent delta slowing, and one patient's EEG was normal. The most commonly used treatment was benzodiazepine and all the patients fully recovered [10-15].

**Table 2:** Seizures and EEG findings in pediatric patients with COVID-19 infection. DOL = day of life.

Authors	Age, sex	Presentation	EEG findings	Treatment	Prognosis
McAbee et al. (2020) [1]	11 years, male	Status epilepticus/encephalopathy	Frontal intermittent delta activity	Not documented	Recovery in six days
Fragoso et al. (2021) [10]	DOL 2, male	Focal to bilateral clonic seizures of the left side	Electrographic seizures	Phenobarbital, midazolam infusion, and steroids	Recovered
Dugue et al. (2020) [11]	6 weeks, male	Cough, fever, and brief episodes of sustained upward gaze associated with bilateral leg stiffening	Excess of sharp temporal transients for age and intermittent vertex delta slowing with normal sleep-wake cycling. No seizures on EEG	None	Recovered
Swarz et al. (2020) [12]	9 years, male	Focal status epilepticus and encephalopathy	Continuous delta slowing throughout the right hemisphere without epileptiform features	Benzodiazepines	Recovery
Shahbaznejad et al. (2020) [13]	18 months, female	Generalized tonic-clonic seizure and status epilepticus with fever	Not documented	Phenobarbital	Recovery
Farley et al. (2020) [14]	8 years, male	Left-sided focal seizure with the rhythmic movement of the left arm and blinking of the left eye	Nonspecific slowing and generalized spike and wave, indicative of diffuse cerebral dysfunction	Benzodiazepine/Keppra 50 mg/kilo	Recovery
Natarajan et al. (2020) [15]	13 years, female	Generalized tonic-clonic seizure, headache, and fever	Normal	Ativan and phenytoin	Recovery

Our review of literature looking for diagnosed epilepsy and association with COVID-19 infection in the pediatric population using PubMed [16,17] showed two patients with known epilepsy with worsening seizures in the setting of COVID-19 infection. One patient was well managed on medication for epilepsy and fully recovered from COVID-19 infection. The other patient had worsening neurological dysfunction after the presentation for worsening epilepsy and COVID-19 infection (Table 3).

**Table 3:** Pediatric patients with a known diagnosis of epilepsy and presentation with COVID-19 infection with outcomes. GTC = generalized tonic-clonic; LoC = loss of consciousness; Bi-PLEDs = bilateral independent periodic lateralized epileptiform discharges.

Authors	Age, sex	Prior diagnosis of epilepsy	Neurological symptoms	Non-neurological symptoms	EEG	Prognosis
Atakla et al. (2020) [16]	14 years, male	Known generalized epilepsy since the age of nine years. Well managed on maintenance Depakote	Worsening GTC seizures, confusion, and LoC	Fever and flu-like symptoms	4-5 Hz theta activity, ample, symmetrical, bilateral, and associated with epileptogenic discharges in the right hemisphere, suggesting multifocal epilepsy	Recovered
Zombori et al. (2021) [17]	17 years, female	Cornelia de Lange syndrome and well-controlled generalized epilepsy	Pediatric multi-inflammatory syndrome temporally related to COVID-19 (PIMS-TS)	Fever	Bi-PLEDs and subclinical seizures	Worsening neurodisability

Epilepsy and seizures are rather rare complications of COVID-19. A systemic review by Dono et al. in 2021 [18] showed 39 articles that reported a total of 47 cases of SE associated with COVID-19 infection. EEG tracings and data were available for 33 (70.2%) patients. Specific documented EEG pattern was described in 14 patients (30.4%), consisting of generalized periodic discharges (GPDs) in 10% of cases and generalized rhythmic delta activity (GRDA) in 4.3% of cases.

Our patient was a new diagnosis of focal epilepsy with cognitive impairment but did not present with any respiratory and other systemic symptoms typical for COVID-19. These spells were reported multiple times a day for several months before the admission, but the EEG read as normal. On admission, there was progression and evolution of spells, which were captured on a routine EEG, and she was diagnosed with focal epilepsy with cognitive impairment. SARS-CoV-2 should also be associated with pediatric patients presenting with primary neurologic symptoms or acute onset of epilepsy.

Understanding of the neurological involvement of the SARS-CoV-2 pathogen COVID-19 in patients with epileptic seizures remains limited. Acute symptomatic seizures can be seen in patients with COVID-19. These seizures are likely to be multifactorial in origin, including cortical irritation due to rupture of the blood-brain barrier, precipitated by the cytokine reaction in viral infection [19]. More information on the characteristics of the clinical course of COVID-19 is needed to better diagnose and treat COVID-19-related neurological symptoms, including seizures, especially in children.

The causes of acute seizures in COVID-19 infection are heterogeneous and can range from metabolic derangement to hypoxia, autoimmune cause, inflammation, etc., but the association of that with a new diagnosis or established diagnosis of epilepsy is not known.

Among the multiple systemic and other environmental factors related to COVID-19 infection causing seizures, the underlying susceptibility for seizures due to epilepsy is likely the most significant factor. Therefore, there is a need for further larger studies and more case reports evaluating COVID-19 in epilepsy patients.

There are case reports in the literature that describe new-onset seizures in severe infection with COVID-19 [5]. However, the data suggest self-limiting provoked seizures in children. There are limited data on the impact of newly diagnosed and or chronic epilepsy in children with COVID-19 infection (Table 3).

## Conclusions

Medical healthcare professionals should be vigilant in assessing for possible seizures, especially in children with systemic effects of COVID-19 infections.

Although data on pediatric cases show a milder disease course than the adults, it is imperative to develop a better characterization and understanding of the pediatric presentation of neurological signs such as seizures or worsening of seizures in chronic epilepsy or recently diagnosed epilepsy with COVID-19 infection for diagnostic purposes and to understand the transmission dynamics in children.
